# A decontamination strategy for resolving SARS-CoV-2 amplicon contamination in a next-generation sequencing laboratory

**DOI:** 10.1007/s00705-022-05411-z

**Published:** 2022-03-17

**Authors:** Peter Mwangi, Milton Mogotsi, Ayodeji Ogunbayo, Teboho Mooko, Wairimu Maringa, Hlengiwe Sondlane, Kelebogile Nkwadipo, Olusesan Adelabu, Philip Armand Bester, Dominique Goedhals, Martin Nyaga

**Affiliations:** 1 Next Generation Sequencing Unit and Division of Virology, Faculty of Health Sciences, University of the Free State, Bloemfontein, 9300 South Africa; 2 Division of Virology, National Health Laboratory Service and University of the Free State, Bloemfontein, South Africa; 3 Pathcare Vermaak, Pretoria, 0157 South Africa

## Abstract

Severe acute respiratory syndrome coronavirus type 2 (SARS-CoV-2) amplicon contamination was discovered due to next-generation sequencing (NGS) reads mapping in the negative controls. Environmental screening was undertaken to determine the source of contamination, which was suspected to be evaporation during polymerase chain reaction (PCR) assays while using the coronavirus disease 2019 (COVID-19) ARTIC protocol. A decontamination strategy is hereby documented to assist laboratories that may experience similar amplicon contamination. Routine molecular laboratory environmental screening as a quality control is highly recommended.

**Supplementary Information:**

The online version contains supplementary material available at 10.1007/s00705-022-05411-z.

## Introduction

Reverse-transcribed and amplified viral sequences (amplicons) are a well-known contamination issue in molecular laboratories [1–5]. Amplicons are generated at a very high copy number during PCR (up to 10^13^ molecules/reaction), posing a significant risk to any molecular investigation due to ensuing carryover contamination [6]. As SARS-CoV-2 research efforts gain unprecedented momentum worldwide, amplicon contamination can prove very disruptive, with precious time and energy diverted to resolving the contamination rather than performing productive research. False-positive results caused by amplicon contamination can jeopardize the efficacy of public health policies, public health responses, surveillance programs, and restriction measures to control the pandemic. The current COVID-19 pandemic has put many molecular diagnostic and research laboratories under exceptional pressure to provide surveillance results, potentially leading to underestimating, ignoring, or even neglecting potential amplicon contamination. Therefore, regular screening for amplicon contamination in the laboratory environment to identify the sources and minimize amplicon contamination is crucial to rule out compromised results. Here, we suggest some useful monitoring and decontamination strategies undertaken after uncovering SARS-CoV-2 amplicon contamination during routine laboratory screening.

## Materials and methods

### Identifying the source of amplicon contamination

After the receipt of extracted SARS-CoV-2 RNA from diagnostic laboratories, the NGS laboratory in context was involved in cDNA generation and whole-genome sequencing. Prior to routine environmental screening, evaporation of amplicons during PCR reactions due to the high denaturation temperature (98 °C) in the ARTIC SARS-CoV-2 sequencing protocol had occurred and was suspected to be the cause of the amplicon contamination. Swabbing of the surfaces in and around the thermocyclers appeared to confirm this, although other potential sources could not be ruled out.

### Procedure for decontamination of environmental surfaces

A decontamination strategy was implemented twice daily for five weeks. First, all the instruments and equipment in the laboratory were covered with sterile plastic bags. Then 75% ethanol (Sigma-Aldrich, St. Louis, MO, USA) was sprayed on the ceiling, walls and in the air and left for 30 minutes. Fresh 0.5% sodium hypochlorite (Sigma-Aldrich, St. Louis, MO, USA) solution was prepared for each use [7] and used to decontaminate the laboratory surfaces such as benches and shelves and left for 30 minutes. Racks were immersed in 0.5% sodium hypochlorite (Sigma-Aldrich, St. Louis, MO, USA) solution for 10 minutes. Afterwards, double-distilled water from a Direct-Q® 3UV Water Purification System (Merck KGaA, Darmstadt, Germany) was used to clean the surfaces, followed by spraying with 75% ethanol (Sigma-Aldrich, St. Louis, MO, USA) and wiping with paper towels. The laboratory equipment (pipettes and thermocyclers) was wiped with absolute ethanol (Sigma-Aldrich, St. Louis, MO, USA) and later with DNA Decontamination Reagent (Merck KGaA, Darmstadt, Germany) according to the manufacturer’s instructions.

### Extraction of RNA

After each decontamination process, swabs were taken from over 15 different parts and surfaces of the laboratory using sterile medical-grade polyurethane swabs (Cleansafe Labs, Cape Town, South Africa). Swabs were stroked in an ‘S’ shape, both vertically and diagonally. The swab was then cut and placed in a 2-ml cryogenic vial (Corning, MA, USA) containing saline buffer solution (Adcock Ingram, Johannesburg, South Africa). Automated RNA extraction was performed using a NUCLISENS EASYMAG instrument (Biomerieux, Marcy I’Etoile, France) as per the manufacturer’s instructions.

### qPCR assay

Primers and probes from a TaqPath™ COVID-19 CE-IVD RT-PCR Kit, targeting three SARS-CoV-2 genes (S, N, and ORF1ab) were utilized to perform a qPCR assay using QuantStudio 7 (Thermo Fisher, Oregon, USA). The limit of detection of the molecular assay was set at a cycle threshold (C_t_) value of 37.

## Results

**Fig. 1:**
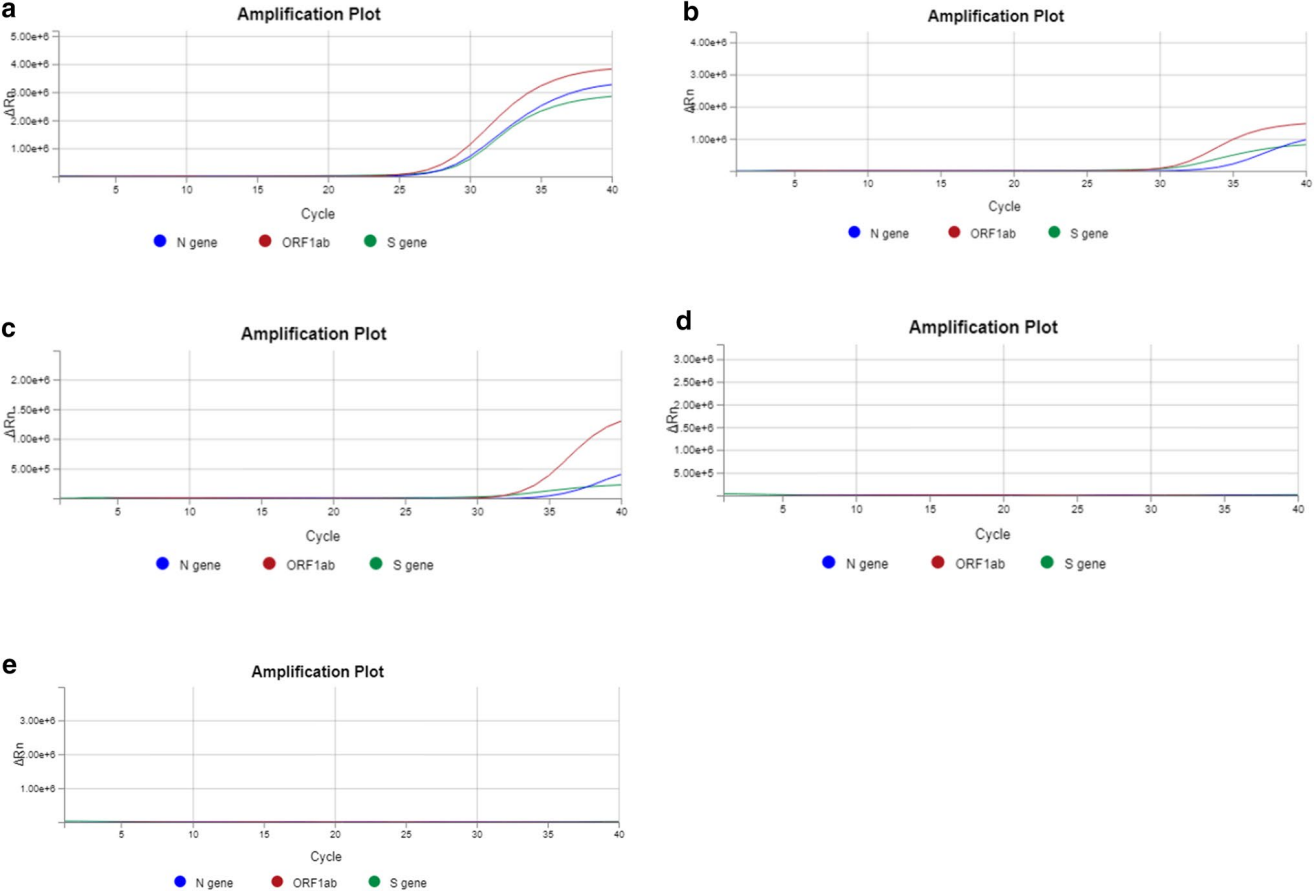
Representative standard qPCR curves indicating the presence/absence of contamination on the swabbed surface general DNA bench. On the *y*-axis, Rn is the fluorescence of the reporter dye divided by the fluorescence of a passive reference dye. In this view, Rn is plotted against the PCR cycle number on the *x*-axis. Three SARS-CoV-2 genes – N, S, and ORF1ab – were targeted for amplicon contamination. Panels a to e represent the first to the fifth week of screening, respectively.

The qPCR reports were recorded to evaluate the effectiveness of the decontamination strategy (Table 1 and Fig. 1). The positive control was a sample from a patient who had tested positive for SARS-CoV-2 with a nadir in C_t_ values in the range of 24.74-28.12, 23.60-27.91, and 23.60-27.79 for the N, S, and ORF1ab gene, respectively. The sensitivity for the S and ORF1ab genes was higher (lower C_t_ values) than for the N gene, potentially due to suboptimally designed RT-PCR primers for the N gene. Elution buffer (QIAGEN, Hilden, Germany) was used as a negative control, and no amplification signal was detected during RT-PCR. To ensure that our cut swabs were contamination-free, we also included a cut swab as an additional negative control, which also did not produce an amplification signal. Amplicons were found at high titers (C_t_ < 37) on several objects and surfaces, including thermocyclers, pipettes, bench surfaces, doorknobs, a laboratory calculator, and PCR cabinets (Table 1). A decreasing trend in amplicon contamination detection was observed over the course of the five weeks of decontamination. Amplicon contamination was still persistent in the fourth week on four of the 19 surfaces that were swabbed (Table 1 and Supplementary Table S1). Intriguingly, we observed fluctuation between positive and negative results on two surfaces: the DNA quantification bench and the outer surface of a -20 °C DNA storage freezer (Table 1). However, after including a DNase decontamination reagent (Sigma-Aldrich, St. Louis, MO, USA) as part of the decontamination routine in the fifth week, we observed notable elimination of the amplicons on all of the swabbed surfaces (Table 1 and Supplementary Table S1). A graphical representation of the real-time PCR data was captured, and fifteen out of the nineteen surfaces (Table 1 and Supplementary Table S1) that were swabbed showed no amplicon contamination by the fourth week (Fig. 1).

**Table 1:** Reported C_t_ values for laboratory surfaces swabbed for SARS-CoV-2 amplicon contamination

Laboratory surface/equipment	Targeted gene	C_t_ value (Week 1)	C_t_ value (Week 2)	C_t_ value (Week 3)	C_t_ value (Week 4)	C_t_ value (Week 5)
General work bench	S	24.60	23.30	28.71	> 37	> 37
	N	26.07	26.62	31.41	> 37	> 37
	ORF1ab	24.83	23.26	> 37	> 37	> 37
Thermocycler	S	26.55	26.04	> 37	> 37	> 37
	N	31.43	35.15	> 37	> 37	> 37
	ORF1ab	29.28	30.96	> 37	> 37	> 37
Thermocycler bench	S	27.73	27.39	> 37	> 37	> 37
	N	35.54	> 37	> 37	> 37	> 37
	ORF1ab	30.25	28.43	> 37	> 37	> 37
PCR cabinet	S	24.85	28.82	28.49	27.82	> 37
	N	28.24	32.54	29.74	31.61	> 37
	OR1ab	27.45	29.63	29.20	30.71	> 37
DNA quantification bench	S	28.51	> 37	24.51	> 37	> 37
	N	36.44	> 37	33.79	> 37	> 37
	ORF1ab	30.95	32.72	28.71	> 37	> 37
Outer surface of -20 DNA storage freezer	S	26.43	27.94	> 37	36.03	> 37
	N	31.07	31.64	35.42	> 37	> 37
	ORF1ab	29.34	29.75	33.17	> 37	> 37
Laboratory calculator in DNA bench	S	19.58	26.53	> 37	> 37	> 37
	N	25.61	32.64	> 37	> 37	> 37
	ORF1ab	20.84	26.98	> 37	> 37	> 37
Controls						
Lab cut swab	S	ND	ND	ND	ND	ND
	N	ND	ND	ND	ND	ND
	ORF1ab	ND	ND	ND	ND	ND
Positive control (positive viral sample)	S	24.56	23.60	27.91	25.46	25.76
	N	27.32	26.43	28.12	27.41	24.74
	ORF1ab	26.01	24.91	27.79	26.82	23.60
Negative control (elution buffer)	S	ND	ND	ND	ND	ND
	N	ND	ND	ND	ND	ND
	ORF1ab	ND	ND	ND	ND	ND

The laboratory surfaces were swabbed for five weeks after the decontamination process. Three SARS-CoV-2 genes – spike (S), nucleoprotein (N), and ORF1ab – were targeted. The limit of detection of the molecular assay was set at a C_t_ value of 37. ND denotes no detection of the amplification signal. The positive control was from a patient who had tested positive for SARS-CoV-2. Elution buffer was used as a negative control. Additional swabbed surfaces and the C_t_ values are provided in Supplementary Table 1.

## Discussion

An effective amplicon decontamination routine using 0.5% sodium hypochlorite, distilled water, 75% ethanol (absolute ethanol for equipment), and DNA decontamination reagent is reported. We screened over 15 different laboratory surfaces, as a small amount of an amplified PCR product that infiltrates laboratory equipment or is present in aerosols can easily spread throughout the lab. While amplicon contamination of surfaces such as benches, shelves, and laboratory equipment such as pipettes and thermocyclers is often easily detectable, it may require looking beyond the obvious to identify other contaminated objects that could easily be overlooked. From our experience, such objects may include items such as laboratory armchairs, calculators, doorknobs, timers, and more importantly, the bottles containing the reagents (ethanol and sodium hypochlorite) used for decontamination. Furthermore, different laboratory surfaces are colonized differently by the amplicons, explaining why it took somewhat longer to fully eliminate amplicons on some surfaces than others.

Any suspicions of co-evaporation of DNA with water during PCR, as has been reported by laboratory practitioners, should be treated with extreme caution. Co-evaporation of DNA with water during PCR has been demonstrated to occur in a previous study, regardless of seal type and pre-heating the thermocycler lids, resulting in cross-contamination arising from migration of DNA from well to well [8]. The use of mineral oil and paraffin wax has been proposed previously as a strategy to avoid false-positive PCR results [9–11]. Alternatively, 8-cap strips can be used as a seal type.

While focusing on decontaminating laboratory surfaces is essential, contaminated reagent kits can be a potential contamination source. Contamination arising from reagent production has been reported recently and has raised concerns [12–14]. Whether contamination occurs during the preparation of reagents or in the laboratory, negative controls provided in the test kit, as well as in-house laboratory controls such as elution buffer and nuclease-free water should show no amplification [15]. Once the kit is opened, reagents should be stored in aliquots in sterile containers. Additionally, careful handling and storage are imperative to prevent contamination of reagent boxes and aliquots. Non-sterile handling with the same gloves used for other laboratory activities should be avoided.

Additionally, personal protective equipment (PPE) should always be worn during laboratory work. When moving from one area of the laboratory to another (e.g., from the pre- to post-PCR area), the full set of PPE should be changed and the workflow strictly adhered to. If possible, different colors of PPE may be used in different areas of the laboratory. Laboratory practitioners and the cleaning staff should be reminded that laboratory guidelines necessitate unidirectional workflow, therefore, they should regard each section of the laboratory as a compartmentalized room to avoid amplicon transfer, especially for non-compartmentalized molecular laboratories, which could be more prone to contamination. Gloves should be sterilized frequently with 70% ethanol and changed when moving against the direction of flow to prevent cross-contamination but preferably changed for each compartment. The PPE should be removed in a manner that avoids contact with external surfaces and disposed of in a dedicated waste container. This should be followed by washing hands with soap and water or sanitizing with 70% alcohol solution. Further guidelines, as provided by WHO for working with SARS-CoV-2 samples, should be followed [16]. It is essential to be cognizant of the potential for amplicon contamination by implementing quality control screening measures. Regular screening of the laboratory environment is essential to monitor amplicon contamination and prevent false-positive results.

## Supplementary Information

Below is the link to the electronic supplementary material.

Supplementary file1 (DOC 70 kb)
