# Supplementary material for: A decontamination strategy for resolving SARS-CoV-2 amplicon contamination in a next-generation sequencing laboratory
Source: Arch Virol. 2022 Mar 17;167(4):1175–9. doi: 10.1007/s00705-022-05411-z (PMC8926888; doi:10.1007/s00705-022-05411-z)
Supplement: Supplementary file 1 — Supplementary file1 (DOC 70 kb) [file 705_2022_5411_MOESM1_ESM.doc]

**Supplementary Table S1** Reported C_t_ values of laboratory surfaces swabbed for SARS-CoV-2 amplicon contamination

| Laboratory surface/equipment | Targeted gene | Ct value (Week 1) | Ct value  (Week 2) | Ct value  (Week 3) | Ct value  (Week 4) | | Ct value  (Week 5) | |
| --- | --- | --- | --- | --- | --- | --- | --- | --- |
| Biodrop screen | S | 26.95 | > 37 | > 37 | > 37 | > 37 | |  |
|  | N | 35.08 | > 37 | > 37 | > 37 | > 37 | |  |
|  | ORF1ab | 31.84 | 31.97 | > 37 | > 37 | > 37 | |  |
| Pipettes in DNA quantification bench | S | 19.12 | 24.67 | 27.76 | 29.21 | > 37 | |  |
|  | N | 26.87 | 28.67 | > 37 | > 37 | > 37 | |  |
|  | ORF1ab | 18.42 | 25.68 | 28.40 | 28.68 | > 37 | |  |
| Outside surface of refrigerator | S | 27.53 | 27.90 | > 37 | > 37 | > 37 | |  |
|  | N | > 37 | > 37 | > 37 | > 37 | > 37 | |  |
|  | ORF1ab | > 37 | > 37 | > 37 | > 37 | > 37 | |  |
| Outside surface of -80°C freezer | S | 24.45 | > 37 | > 37 | > 37 | > 37 | |  |
|  | N | 29.98 | > 37 | > 37 | > 37 | > 37 | |  |
|  | OR1ab | 27.31 | > 37 | > 37 | > 37 | > 37 | |  |
| Pipettes in the general work bench | S | 20.79 | 28.98 | > 37 | > 37 | > 37 | |  |
|  | N | 24.83 | > 37 | > 37 | > 37 | > 37 | |  |
|  | ORF1ab | 21.81 | > 37 | > 37 | > 37 | > 37 | |  |
| Heat block | S | 25.53 | > 37 | > 37 | > 37 | > 37 | |  |
|  | N | 34.25 | > 37 | > 37 | > 37 | > 37 | |  |
|  | ORF1ab | 29.23 | > 37 | > 37 | > 37 | > 37 | |  |
| Office doorknob | S | 29.12 | 29.82 | > 37 | > 37 | > 37 | |  |
|  | N | > 37 | > 37 | > 37 | > 37 | > 37 | |  |
|  | ORF1ab | > 37 | 29.75 | > 37 | > 37 | > 37 | |  |
| Outer surface of a -20 freezer | S | 26.43 | 27.94 | > 37 | > 37 | > 37 | |  |
|  | N | > 37 | > 37 | > 37 | > 37 | > 37 | |  |
|  | ORF1ab | 29.34 | 29.75 | > 37 | > 37 | > 37 | |  |
| Storeroom door | S | 27.75 | > 37 | > 37 | > 37 | > 37 | |  |
|  | N | > 37 | > 37 | > 37 | > 37 | > 37 | |  |
|  | ORF1ab | 27.18 | > 37 | > 37 | > 37 | > 37 | |  |
| Outer surface of ultrapurifier instrument | S | > 37 | > 37 | - | - | - | |  |
|  | N | > 37 | > 37 | - | - | - | |  |
|  | ORF1ab | > 37 | > 37 | - | - | - | |  |
| Outer surface of an old reagent box | S | 23.09 | 28.22 | > 37 | > 37 | > 37 | |  |
|  | N | 32.08 | 33.92 | > 37 | > 37 | > 37 | |  |
|  | ORF1ab | 25.84 | 31.01 | > 37 | > 37 | > 37 | |  |
| Arm supports of two chairs in the lab | S | - | - | - | 27.74 | > 37 | |  |
|  | N | - | - | - | > 37 | > 37 | |  |
|  | ORF1ab | - | - | - | 27.18 | > 37 | |  |

The laboratory surfaces were swabbed for five weeks after the decontamination process. Three SARS-CoV-2 genes – spike (S), nucleoprotein (N) and ORF1ab –were targeted. The limit of detection of the molecular assay was set at a C_t_ value of 37. ND denotes no detection of the amplification signal. The weeks when swabbing was not performed are denoted by a dash (-) symbol.
